# Giving birth during a pandemic: From elation to psychopathology

**DOI:** 10.1002/ijgo.13803

**Published:** 2021-07-14

**Authors:** Jose A. Puertas‐Gonzalez, Carolina Mariño‐Narvaez, Borja Romero‐Gonzalez, Maria Isabel Peralta‐Ramirez

**Affiliations:** ^1^ Mind, Brain and Behaviour Research Center (CIMCYC) Granada Spain; ^2^ Personality, Assessment and Psychological Treatment Department Faculty of Psychology University of Granada Granada Spain; ^3^ Psychology Department Faculty of Education Campus Duques de Soria University of Valladolid Valladolid Spain

**Keywords:** anxiety, COVID‐19, depression, postpartum, psychopathology, stress

## Abstract

**Objective:**

To compare the postpartum psychopathological symptoms of women who gave birth before the pandemic with those who gave birth during the pandemic.

**Methods:**

A total of 212 women participated in the study, of which 96 gave birth before the pandemic and 116 during the pandemic. Psychopathological symptoms, postpartum depression, perceived stress, and resilience were evaluated.

**Results:**

Women who gave birth during the pandemic had higher scores on somatization, obsessions and compulsions, interpersonal sensitivity, depression, anxiety, hostility, phobic anxiety, and psychoticism. In addition, perceived stress was the common predictor of an increase in these symptoms.

**Conclusion:**

Postpartum is a complicated period in a woman's life. Many psychological adaptations take place and women may be subject to psychological alterations during this period. In addition, women who gave birth during the COVID‐19 crisis may show greater psychological vulnerability, due to the specific situation experienced during the pandemic. The COVID‐19 pandemic may have played a role in the increase in psychopathological symptoms after childbirth. Detecting possible symptoms postpartum plays a crucial role, because it allows intervening and preventing the development of psychopathologies.

## Synopsis

Women who gave birth during the pandemic had higher scores for somatization, obsessions and compulsions, interpersonal sensitivity, depression, anxiety, hostility, phobic anxiety, and psychoticism.

## 1 INTRODUCTION

Postpartum brings about many transformations in a woman's life: changes in hormones and in the brain, as well as social and psychological modifications. One major change takes place in the brain: to ensure the survival and care of the newborn, the brain increases its functional and structural plasticity.^1^ As a result, it has been discovered that the parts of women's brains related to the detection of threats, as well as emotional recognition, are more active in the postpartum period.^2^

These adjustments in the brain are associated with emotional transformations, which make postpartum women more susceptible to psychological alterations. Postpartum is thus a critical period for the detection of such changes and for the prevention of psychopathological disorders.^3^

In addition, it has also been found that before delivery, pregnant women show more symptoms of somatization, phobic anxiety, or paranoid ideation than non‐pregnant women.^4^ These results suggest that mothers possibly undergo some form of “psychological vulnerability” both during pregnancy and postpartum.^1^

In a similar line, other studies have shown that events experienced during pregnancy and postpartum may be the precursors of more serious diseases.^5^

Traumatic or unusual experiences, such as the current global COVID‐19 pandemic, may encourage the development of psychological disorders. In the light of this historical event, the psychological impact of the crisis on the population has been an object of study. It has been observed that, due to fear of contagion, the symptoms of anxiety and levels of stress have considerably risen among the population.^6^

In the case of pregnant women, higher levels of stress, anxiety, and depression have been found as a result of being exposed to the stressful ordeal of living through a pandemic.^7^ In the same way, various studies have explored symptoms of postpartum depression in women who gave birth during the pandemic and higher levels of postpartum depression were found.^8^ However, these studies did not perform an in‐depth examination of the wide range of psychopathological symptoms that women may present after childbirth.

Given the psychological repercussions of both giving birth and experiencing the pandemic, it has become more essential to study the psychological health of women. Therefore, the aim of the present study was to verify the psychological and emotional state of women who gave birth during the pandemic compared with that of women who gave birth before it. The psychopathological symptoms of women who gave birth before the pandemic were compared with those who gave birth during quarantine. Possible variables related to these symptoms were also verified.

## 2 MATERIALS AND METHODS

### 2.1 Participants

A total of 240 women (mean age 33.46 ± 4.35 years) were recruited and consented to take part in the study. Of them, 212 women finally participated in the present study (13 women did not fill in the questionnaire, 10 filled in the questionnaire more than 1 month after delivery, and five women had been diagnosed with one physical or psychological disease in the previous year). The inclusion criteria were as follows: to be of legal age; to have given birth at some point before or after the State of Emergency was decreed in Spain; to complete the questionnaire within the first month after delivery; to read and write in Spanish; and to have an Internet connection. The exclusion criterion was experiencing physical or mental illness at the time of being diagnosed with any of them in the previous year.

All participants gave prior consent before being included in the study. Participation was voluntary and the study was conducted in accordance with the Declaration of Helsinki (World Medical Association, 2013) and the European Union Good Clinical Practice Directive (Directive 2005/28/EC). The study protocol was approved by the University of Granada's Human Research Ethics Committee (reference code 1580/CEIH/2020 and reference number 881).

### 2.2 Instruments

First, sociodemographic and related variables for birth and newborns were collected from participants. In addition, a psychological assessment was performed using the assessment tools listed below.

- The Symptom Checklist‐90‐Revised (SCL‐90‐R).^9^ This tool measures nine dimensions of psychopathological symptoms (somatization, obsessive‐compulsive, interpersonal sensitivity, depression, anxiety, hostility, phobic anxiety, paranoid ideation, and psychoticism) and three global indices of psychological distress. The Cronbach's alpha reliability coefficients of the Spanish version is in the range of 0.67<α<0.94.^10^
- Perceived Stress Scale (PSS).^10^ This scale assesses the degree to which people find their lives unpredictable, uncontrollable, or overcharged in the previous month. The Spanish version of the PSS (14 items) showed a high internal consistency at 0.81.
- Connor–Davidson Resilience Scale (CD‐RISC).^11^ The CD‐RISC reflects the ability to resist experiences such as change, personal problems, illness, pressure, failure, and feelings of pain. The Cronbach alpha confidence factor for CD‐RISC‐10 is 0.85.
- Edinburgh Postnatal Depression Scale (EPDS).^12^ The EPDS is used to assess the risk of postpartum depression. Its reliability, in terms of internal consistency, is acceptable (α = 0.79).

In addition, in the group of women who gave birth during pandemic, the following assessment tool was used: Birth Satisfaction Scale‐Revised (S‐BSS‐R).^13^ Three subscales measured overall satisfaction with childbirth: stress during childbirth; personal attributes; and quality of care. The instrument presented adequate internal reliability (α = 0.77).

### 2.3 Procedure

First, before the pandemic, and coinciding with the Gestastress research protocol, women who had just given birth in Spanish hospitals were recruited. These women, who gave birth between March 2019 and February 2020, constituted the group of women who gave birth before the pandemic. The procedure for recruiting these women was as follows: they were informed of the study after giving birth, and those who agreed to participate filled in the informed consent form. At this point, the questionnaires were sent to them online, using Google forms (sociodemographic variables, SCL‐90‐R, PSS, EDPS, and CD‐RISC) and they were given a maximum of 1 month to fill them in, according to the inclusion criteria.

Second, after the sudden onset of the pandemic, and following the declaration of a State of Emergency by the Spanish Government, participants continued to be recruited. However, those women who agreed to participate (through the procedure explained above) were included in the group of women who gave birth during the pandemic (from April 1, 2020, to July 1, 2020).

This left two groups (women who gave birth before the pandemic vs those who gave birth during the pandemic). The data obtained and the differences between the two groups were analyzed.

### 2.4 Data analysis

A descriptive analysis (mean ±standard deviation [SD]) was performed of the sample's main continuous variables, of a sociodemographic and obstetric nature. A frequency analysis was carried out for the remaining categorical variables.

In order to verify whether there were any significant differences in psychopathological symptoms between women who gave birth before and during the pandemic, a Student t‐test was performed. The dependent variables were the scores of the nine main dimensions of the SCL‐90‐R and postpartum depression score, and the independent variable was the moment of delivery (before or during the pandemic). Whether the woman was primiparous or not was included as a covariate.

Finally, to verify which psychological variables could predict psychopathological symptoms in women who gave birth during the pandemic, various hierarchical linear regression analyses were conducted, the dependent variables being the scores on the SCL‐90‐R subscales. The variables related to delivery were included in step 1 (gestational age at birth, whether primiparous or not, vaginal or instrumental delivery, and satisfaction with delivery), and in step 2, the predictive variables were the perceived stress and resilience (PSS, CD‐RISC) scores. A collinearity diagnosis was performed to examine variable associations. Tolerance greater than 0.3 and a variance inflation factor below 10 indicate the absence of multicollinearity.

The analyses were carried out using the SPSS version 26.0 (IBM Corp., Armonk, NY, USA).

## 3 RESULTS

### 3.1 Sample description

The total sample of participants was divided into two groups: the first group consisted of 96 women (mean age 32.96 ± 3.97 years) who gave birth in the pre‐pandemic period; and the second group consisted of 116 women (mean age 33.86 ± 4.60 years) who gave birth during the pandemic.

Both groups were found to be even in relation to the main sociodemographic and obstetric history variables. Statistically significant differences were found between both groups, however, regarding whether they were primiparous or multiparous (ꭓ^2^ = 5.62; *P* = 0.018) (Table 1).

**TABLE 1:** Description and comparison in sociodemographic variables and obstetric history, in women who gave birth before and during the pandemic.^a^

	Before pandemic (n=96)	During pandemic (n=116)	t/ꭓ^2^	*P*
Sociodemographic variables				
Age (years)	32.96 ± 3.97	33.86 ± 4.60	0.46	0.13
Nationality				
Spanish	77 (83.7)	86 (74.1)	2.76	0.09
Immigrant	15 (16.3)	30 (25.9)		
Marital status				
Married/cohabiting	91 (98.9)	109 (94)	3.39	0.06
Single/widow	1 (1.1)	7 (6)		
Level of education				
No school	1 (1.1)	—	1.49	0.68
Primary school	1 (1.1)	2 (1.7)		
Secondary school	17 (18.7)	20 (17.2)		
University	72 (79.1)	94 (81)		
Obstetric information				
Nulliparous				
Yes	46 (50)	75 (66.4)	5.62	0.018^b^
No	46 (50)	38 (33.6)		
Delivery				
Vaginal	63 (64.3)	77 (66.4)	0.103	0.748
Instrumental	35 (35.7)	39 (33.6)		
Sex of infant				
Male	43 (44.8)	59 (50.9)	0.77	0.37
Female	53 (55.2)	57 (49.1)		
Gestational age (weeks)	39.48 ± 1.34	39.06 ± 1.79	2.77	0.06
Birth weight (g)	3283.39 ± 462.76	3228.19 ± 484.31	0.44	0.39

^a^ Values are given as number (percentage) or mean ± SD, unless otherwise specified. In some variables there are missing values, so N may not correspond to the corresponding one for each group.

^b^ Significance at *P* < 0.01.

### 3.2 Differences in psychopathological symptoms and postpartum depression between women who gave birth before and during the pandemic

Mean comparison analyses showed statistically significant differences between the pre‐pandemic group of women and the group of women who gave birth during the pandemic in most SCL‐90‐R subscales, specifically for somatization, obsessions and compulsions, interpersonal sensitivity, depression, anxiety, hostility, phobic anxiety, and psychoticism. In addition, all scores were higher in the group of women who gave birth during the pandemic, notably regarding obsessions and compulsions, presenting clinical scores over 70. The results are shown in Table 2.

**TABLE 2:** Differences in psychopathological symptoms among women who gave birth before and during the pandemic.^a^

	Before pandemic (n=96)	During pandemic (n=116)	*F*	*P*
SCL−90‐R				
Somatization	46.54 ± 27.19	59.86 ± 26.84	11.24	0.001^b^
Obsessions and compulsions	46.71 ± 32.43	72.39 ± 28.06	33.52	0.000^b^
Interpersonal sensitivity	40.18 ± 34.87	62.82 ± 32.37	21.92	0.000^b^
Depression	41.47 ± 30.06	66.12 ± 30.36	32.31	0.000^b^
Anxiety	39.74 ± 30.42	62.20 ± 29.68	26.52	0.000^b^
Hostility	42.48 ± 33.04	55.79 ± 32.84	7.79	0.006^b^
Phobic anxiety	40.97 ± 36.01	60.14 ± 36.76	12.05	0.001^b^
Paranoid ideation	40.49 ± 36.31	48.65 ± 36.64	2.53	0.113
Psychoticism	45.33 ± 37.37	57.74 ± 37.14	5.24	0.023^c^
EPDS	7.78 ± 4.75	7.70 ± 5.42	0.000	0.983

Abbreviations: EPDS, Edinburgh Postnatal Depression Scale; SCL‐90‐R, Symptom Checklist‐90‐Revised.

^a^ Values are given as mean ± SD, unless otherwise specified.

^b^ Significance at *P* ≤ 0.01.

^c^ Significance at *P* ≤ 0.05.

No differences were found for postpartum depression, with 25 women (26%) displaying depressive symptomatology (score >10 in EPDS) in the pre‐pandemic group, compared to 33 women (28.4%) in the group who gave birth during the pandemic.

### 3.3 Predictive psychological variables of psychopathological symptoms in women who gave birth during the pandemic

Psychological variables were analyzed as predictors of psychopathological symptoms, using hierarchical linear regression analysis. All models were significant after controlling for variables related to delivery (gestational age at birth, type of delivery, primiparous or not, and satisfaction with delivery). Furthermore, perceived stress was the only predictor variable for the following psychopathological symptoms: somatization; obsession and compulsion; interpersonal sensitivity; anxiety; phobic anxiety; and psychoticism. In the case of depression, resilience was found as a predictor, along with perceived stress. Regarding hostility, the two predictors were perceived stress and being primiparous. These data are shown in Table 3.

**TABLE 3:** Hierarchical linear regression analyses for psychological variables as predictors of psychopathological symptoms.

	β	*P*	R‐square	Increased R‐square	F
Model 1 Dependent variable: Somatization					
Block 1					
Gestational age	0.100	0.281	0.080	0.080	2.345
Delivery^a^	0.036	0.712			
Primiparous	−0.012	0.906			
S‐BSS‐R	−0.259	0.010			
Block 2					
Gestational age	0.127	0.127	0.254	0.174	6.022^b^
Delivery^a^	0.031	0.732			
Primiparous	0.026	0.774			
S‐BSS‐R	−0.062	0.533			
Perceived stress	0.453	0.000^b^			
Resilience	−0.070	0.413			
Model 2 Dependent variable: Obsessions and compulsions					
Block 1					
Gestational age	−0.026	0.780	0.079	0.079	2.314
Delivery^a^	−0.038	0.698			
Primiparous	−0.087	0.374			
S‐BSS‐R	−0.295	0.003			
Block 2					
Gestational age	0.010	0.907	0.285	0.206	7.045^b^
Delivery^a^	−0.039	0.661			
Primiparous	−0.054	0.543			
S‐BSS‐R	−0.074	0.443			
Perceived stress	0.503	0.000^b^			
Resilience	−0.015	0.859			
Model 3 Dependent variable: Interpersonal sensitivity					
Block 1					
Gestational age	0.018	0.846	0.106	0.106	3.194^c^
Delivery^a^	0.135	0.226			
Primiparous	−0.117	0.165			
S‐BSS‐R	−0.279	0.005			
Block 2					
Gestational age	0.059	0.463	0.306	0.238	9.235^b^
Delivery^a^	0.137	0.106			
Primiparous	−0.084	0.320			
S‐BSS‐R	−0.041	0.659			
Perceived stress	0.542	0.000^b^			
Resilience	0.010	0.902			
Model 4 Dependent variable: Depression					
Block 1					
Gestational age	0.031	0.741	0.091	0.091	2.695^c^
Delivery^a^	0.100	0.306			
Primiparous	−0.109	0.266			
S‐BSS‐R	−0.272	0.006			
Block 2					
Gestational age	0.066	0.363	0.464	0.374	15.321^b^
Delivery^a^	0.089	0.245			
Primiparous	−0.050	0.512			
S‐BSS‐R	0.011	0.894			
Perceived stress	0.653	0.000^b^			
Resilience	−0.141	0.049^c^			
Model 5 Dependent variable: Anxiety					
Block 1					
Gestational age	−0.043	0.643	0.087	0.087	2.566^c^
Delivery	0.029	0.521			
Primiparous	−0.063	0.764			
S‐BSS‐R	−0.292	0.003			
Block 2					
Gestational age	−0.009	0.909	0.334	0.247	8.861^b^
Delivery^a^	0.024	0.815			
Primiparous	−0.020	0.775			
S‐BSS‐R	−0.055	0.554			
Perceived stress	0.543	0.000^b^			
Resilience	−0.068	0.397			
Model 6 Dependent variable: Hostility					
Block 1					
Gestational age	−0.075	0.417	0.100	0.100	3.004^b^
Delivery^a^	0.051	0.597			
Primiparous	−0.218	0.026			
S‐BSS‐R	−0.273	0.006			
Block 2					
Gestational age	−0.051	0.547	0.278	0.177	6.786^b^
Delivery^a^	0.043	0.624			
Primiparous	−0.177	0.047^c^			
S‐BSS‐R	−0.078	0.424			
Perceived stress	0.450	0.000^b^			
Resilience	−0.097	0.249			
Model 7 Dependent variable: Phobic anxiety					
Block 1					
Gestational age	−0.093	0.329	0.042	0.042	1.179
Delivery^a^	0.041	0.686			
Primiparous	−0.027	0.789			
S‐BSS‐R	−0.170	0.092			
Block 2					
Gestational age	−0.063	0.480	0.173	0.131	3.695^b^
Delivery^a^	0.041	0.663			
Primiparous	−0.001	0.991			
S‐BSS‐R	0.006	0.951			
Perceived stress	0.402	0.000^b^			
Resilience	−0.002	0.985			
Model 8 Dependent variable: Psychoticism					
Block 1					
Gestational age	−0.065	0.482	0.091	0.091	2.703^c^
Delivery^a^	0.078	0.407			
Primiparous	−0.081	0.424			
S‐BSS‐R	−0.276	0.005			
Block 2					
Gestational age	−0.028	0.734	0.312	0.221	7.999^b^
Delivery^a^	0.078	0.366			
Primiparous	−0.046	0.592			
S‐BSS‐R	−0.048	0.614			
Perceived stress	0.521	0.000^b^			
Resilience	−0.012	0.883			

Abbreviations: S‐BSS‐R, Birth Satisfaction Scale Revised.

^a^ Delivery (vaginal or instrumental).

^b^ Significance at *P* < 0.01.

^c^ Significance at *P* < 0.05.

## 4 DISCUSSION

The aim of the present study was to verify the psychological and emotional status of women who have given birth during a pandemic. To do this, it was first verified whether any differences could be found in a range of psychopathological symptoms between women who had given birth before and during the pandemic. A check was subsequently made as to whether birth and psychological variables were related to, or acted as, predictors of such psychopathological symptoms.

The results of the present study indicate that women who had given birth during the pandemic presented more psychopathological symptoms than those who had given birth during the pre‐pandemic period, specifically somatization, obsessions and compulsions, interpersonal sensitivity, anxiety, depression, hostility, phobic anxiety, and psychoticism.

Some authors have shown that, during this pandemic, women consider postpartum as a period that involves even more challenges than before; this perception can lead to psychological stress and make women more vulnerable to emotional disturbances. It can also reduce their psychological well‐being.^14^ In addition, various studies have shown that levels of stress and depression increased during pregnancy as a result of the pandemic.^7^, ^15^ Moreover, a woman's concerns about the pandemic and everything around her, such as fear of contagion, being alone during childbirth, or possible vertical transmission to the fetus, simply contribute to the development of these symptoms of anxiety.^7^

Other psychopathological symptoms such as obsessions, compulsions, and phobic anxiety are equally more widespread in the current situation, characterized by excessive hygiene and fear of contagion.^16^ The latter, coupled with the fact that during postpartum, the likelihood of experiencing obsessions and compulsions increases twofold, could explain the differences found and the growth in symptomatology.^17^ However, it should be noted that the scores found in the present study exceeded, on average, the 70th percentile. This latter psychopathological dimension thus presented clinical scores, with all its therapeutic implications.

As far as somatization is concerned, this increased during pregnancy, due to the complex emotional and physiological processes proper to gestation.^4^ Although the role of postpartum somatization is currently unknown, it has been found to increase in different samples of the population. Therefore, it would be unsurprising if it was found to increase after childbirth as a result of the pandemic.^18^

Finally, symptoms such as interpersonal sensitivity, hostility, and psychoticism can be explained by the social restrictions imposed on the population.^9^ The pandemic's side effects include a rise in individualism and loneliness derived from a halt to social relations. These latter side effects may lead to the accentuation of this type of symptomatology.^19^ In addition, the pregnancy's own evolutionary perspective means that the mother is capable of detecting threats in the environment to protect her infant and will reject anyone outside her “group,” which may exacerbate the symptoms described at the same time.^4^, ^20^

As far as postpartum depression is concerned, no difference was found between the two groups in the present study. One possible explanation is that this problem presents a high incidence during postpartum anyway, making it unlikely to detect differences in symptomatology.^21^ Some authors have reported a rise in these symptoms of depression.^22^ However, the failure to find any differences demonstrates that women are vulnerable to postpartum depression no matter the moment during which it is experienced, and the first signs of the disease must be addressed.

Most of these psychopathological symptoms, which were more widespread in women who gave birth during the pandemic, shared a common predictor variable: that of perceived stress. Stress is one of the psychological problems that has increased the most during this pandemic. This is unsurprising, because the unpredictability of the situation added to a sense of low personal control over it, leading to increased levels of stress.^23^ In addition, other authors have found that during pregnancy, perceived stress is also a predictor of psychopathology.^4^ Resilience, an important pregnancy variable, equally seems to be a possible predictor and could dampen the severity of psychopathological symptoms.^24^ This is a significant finding because it makes it possible to focus on the levels of perceived stress when preparing interventions directed towards the psychological health of a pregnant woman and the postpartum stage.

A limitation of this study is the fact that the sample was exclusively composed of Spanish women. This restricts the possibility of generalizing the results, as they can only be attributed to women who experienced the pandemic in Spain. Nevertheless, given the pandemic's global nature, similar results are likely be found in other countries.

In conclusion, the results of the present study highlight the delicate period women endure around childbirth, and such conditions have been aggravated by a pandemic. The COVID‐19 pandemic has affected the whole world, and these women, who must go through the important life process of childbirth, have increased psychopathological symptoms. As a result, it has become essential to work on psychopathological symptoms during pregnancy, to alleviate the effects and their exacerbation after childbirth. Especially now, when they live in a world that generates uncertainty and fear. In addition, assessing the perceived stress that women experience constitutes an essential step. Indeed, early detection leads to timely intervention and provides women the support and tools they need to help them cope.

## CONFLICTS OF INTEREST

The authors have no conflicts of interest.

## AUTHOR CONTRIBUTIONS

JAP‐G: Conceptualization, methodology, formal analysis, writing the original draft, review and editing the manuscript. CM‐N: Conceptualization, methodology, data curation. BR‐G: Conceptualization, data curation, formal analysis, methodology. MIP‐R: Funding acquisition, conceptualization, review and editing the manuscript, supervision.
